# Extreme oncoplasty: past, present and future

**DOI:** 10.3389/fonc.2023.1215284

**Published:** 2024-01-30

**Authors:** René Aloisio da Costa Vieira, Regis Resende Paulinelli, Idam de Oliveira-Junior

**Affiliations:** ^1^ Postgraduate Program in Tocogynecology, Botucatu School of Medicine, Botucatu, SP, Brazil; ^2^ Postgraduate Program in Oncology, Barretos Cancer Hospital, Barretos, SP, Brazil; ^3^ Department of Surgical Oncology, Division of Breast Surgical Oncology, Muriaé Cancer Hospital, Muriaé, MG, Brazil; ^4^ Department of Gynecology and Obstetrics, University of Goiás, Goiania, GO, Brazil; ^5^ Department of Mastology and Breast Reconstruction, Barretos Cancer Hospital, Barretos, SP, Brazil

**Keywords:** breast neoplasms, oncoplastic surgery, extreme oncoplasty, breast conserving therapy, surgical procedures, surgical flaps

## Abstract

Breast surgery has evolved from mastectomy to breast-conserving surgery (BCS). Breast oncoplastic surgery later emerged with the inclusion and development of techniques used in plastic surgery for breast neoplasms. Recently, a new paradigm has been considered for mastectomy candidates with large multifocal and multicentric tumours, designated extreme oncoplasty (EO), which has allowed new techniques to be applied to tumours that would have been ineligible for BCS before. There are few publications and no uniform descriptions grouping all the technical possibilities and new indications together. We performed this a review with the objective of evaluating the indications and surgeries performed in the EO context, representing a new perspective for BCS. We observed new indications as extensive microcalcifications, locally advanced breast carcinoma with partial response to chemotherapy, small to moderate-sized non-ptotic central tumours and extreme ptosis. Small breasts are able for EO since the presence of ptosis. New surgeries are reported as disguised geometric compensation, perforators flaps, local/regional flaps, latissimus dorsi miniflap and partial breast amputation. It is important to decrease barriers to oncoplastic surgery if we want to increase the use of EO and BCS rates.

## Introduction

Oncoplastic surgery (OS) allows for higher levels of care in breast-conserving surgery (BCS). BCS was initially advised for the treatment of tumours up to 3–5 cm with a favourable breast/tumour ratio, being deemed safe and having an acceptable recurrence rate (1, 2). OS associated with BCS evolved from breast remodelling (3, 4), causing a loss of 20–50% of the breast parenchyma, to the mammoplasty and mastopexy techniques, which was classified as a type II procedure for the above tumours (5, 6). OS was later used for tumours up to 5 cm or multicentric/multifocal tumours, in which case it was designated extreme oncoplasty (EO) (7).

EO is a group of new BCS techniques for patients who are initial candidates for mastectomy. This new paradigm for BCS includes diverse techniques. Recently, a systematic review article described geometric compensation (GC)/split reduction based on Wise pattern (WP) mammoplasty, but it only reviewed one technique that fell under EO (8), noting the improvement of the initial indications. We aimed to review the EO concepts in more detail to summarize the state of the art and propose future directions.

## Materials and methods

A review was conducted to evaluate the indications and surgeries performed in the context of EO. We used the PICO system for article evaluation: Problem = breast neoplasm; Intervention =EO; Comparison = all; Outcome = indication and type of surgery. We also considered OS and reconstructive surgical procedures to find associated surgeries related to EO.

Based on the concept of EO with resection for tumours larger than 5 cm or multicentric/multifocal tumours, and referring to previous publications, we based our search strategy on the following search terms: extreme oncoplasty, geometric compensation, regional flaps and mammaplasty. A review was performed by screening two databases (PubMed and LILACS). To evaluate articles in PubMed, we used the following terms: (((“breast neoplasms”[Mesh]) AND (“surgery, plastic”[Mesh] OR “plastic surgery procedures”[Mesh] OR “mammaplasty”[Mesh] OR “mastectomy, segmental”[Mesh])) AND (“oncoplastic surgery” OR “oncoplasty” OR “oncoplastic” OR “extreme oncoplasty” OR “extreme oncoplastic” OR “regional flaps” OR “geometric compensation”)). The terms used in LILACS were “neoplasias da mama” and “ procedimentos cirúrgicos reconstrutivos”; “neoplasias da mama” and “ cirurgia oncoplastica ou oncoplastia.” The deadline for article publication was 12/31/2022. There was no language restriction. Two authors (RACV, I-OJr) performed the revision and jointly evaluated the full articles selected. Each article selected was evaluated based on the type of study, its main endpoints (**Table 1**; **Supplementary Table 1**) and indications related to EO (**Table 2**). Based on main endpoints we group variables to be considered in future studies (**Supplementary Table 2**). The quality of the studies was evaluated using the MINORS instrument (24) (**Supplementary Table 3**) and NOS (Newcastle-Ottawa Scale) instrument (25) (**Supplementary Table 4**).

**Table 1:** Main studies reporting Extreme Oncoplasty*.

Author (ref)	Year	Number of patients	Type of study	Endpoint
Silverstein (7)	2014		Viability	Options
Paulinelli (9)	2014	17	Descriptive; CG	Clinic results, cosmesis
Silverstein (10)	2015	–	Conceptual; Case-control	Conceptual EO
Silvestein (11)	2016	–	Descriptive	Evolution of EO
Acea Nebril (12)	2017	33 EO 171 control	Case-control	PS, quality of life
Crown (13)	2019	111	Casuistry	PS, techniques, complications
Koppiker (14)	2019	39	Casuistry	PS, techniques, complications, quality of life
Pearce (15)	2020	90	Case-control	PS, techniques, complications recurrence; Subgroup analysis
Paulinelli (16)	2020	73	Descriptive; CG	PS, techniques, complications, follow-up, cosmesis
Savioli (17)	2021	50	Casuistry	PS, techniques, complications
Alder (18)	2021	–	Conceptual	Inclusion of miniflap
Nigram (19)	2021	4	Case series	Inclusion of perforating vessels
Joukainen (20)	2021	98	Casuistry	Imaging
Cakmak (21)	2021	–	Conceptual	Evolution of EO
Paulinelli (22)	2021	29	Disguised CG	Clinic results, cosmesis
De Lorenzi (23)	2022	100	Case-control	Recurrence and survival
Franca (8)	2022	34 + 243 (review)	Casuistry and literature review	Clinic results, cosmesis, literature review

CG, Geometric compensation; EO, extreme oncoplasty; PS, patient selection.

* Summary of the main endpoints of case-control or observational studies is reported in the **Supplementary Table 1**.

**Table 2:** Extreme oncoplasty: Indications and surgeries.

	Indication	Type of Surgery
Classical	Tumour > 5cm	Wyse Pattern
	Multicentric and multifocal tumours	Geometric compensation
	Initial candidates for mastectomy	
	Breast/tumour unfavourable ratio	Modified mammoplasty
New indications	Breast/tumour unfavourable ratio	Modified mammoplasty
	Extensive CDIS or microcalcifications	–
	New or recurrence in irradiated breasts	–
	Locally advanced breast carcinoma with partial response to chemotherapy	–
	Inappropriate scare	–
	Medium and low breast with ptosis	Geometric compensation
New situations	Small to moderate-sized non-ptotic with centrally located breast cancer	Perforators flaps
	Small to moderate sized-breast	Regional volume replacement
	Random	Local/regional flaps
	Pedicle flap	Pedicle flap
	–	Latissimus dorsi miniflap
	Extreme ptosis	Partial breast amputation

## Results

Initially, 806 articles were identified from the PubMed database and 2 articles from LILACS. All articles identified and selected were in English. The titles and abstracts were evaluated, and 140 articles were selected for reading. After content evaluation, 46 articles were selected for this study. For EO specifically, 23 original articles and four comments were evaluated. **Supplementary Figure 1** shows the PRISMA flowchart.

Silverstein suggested the term “EO” and the articles selected here (7, 10, 11). Paulinelli considered the term “GC using WP resection” (9, 16), and similar articles were selected based on modified techniques (22), case descriptions (26–28), and one institutional casuistic with systematic reviews (8). We found articles related to preoperative care (15, 20, 29–31), traditional indications (13, 17), multicentric/multifocal tumours (14, 23), increased indications (8, 13, 19, 21), casuistic (14, 17), and case reports (27, 32, 33). Quality of life (12, 14) was also evaluated. In addition, four replies were found (29–31, 34).

Evaluating the quality of the studies MINORS (**Supplementary Table 3**) score range of 12 to 19 points, and NOS (Newcastle-Ottawa Scale) evaluation (**Supplementary Table 4**) range of 3 to 7 stars. Most studies are retrospective. The best methodological study was a matched case-control study comparing EO with mastectomy (23). Four case−control studies, evaluated level II oncoplastic procedures (10, 12, 23), the indications and surgeries were different and one study performed matched evaluation (23) and one compare volume replacement with latissimus dorsi flaps (15). Of the case-control studies, the comparisons patients who underwent OE or not (10, 12, 23). Two studies present a retrospective component and prospective collection of information such as cosmesis assessment photos (8) and quality of life questionnaires (14). Two studies are prospective, showing the geometric compensation technique and its variation (9, 22). In most studies, follow-up time was short, limiting the assessment of local recurrence. Follow-up is stated in a generic way, without description related to patients’ loss of follow-up, being considered positive in studies with cosmesis and with quality outcomes.

EO was associated with higher tumour size, higher specimen weight, narrower margins, and possible conversion to mastectomy, without increasing the recurrence rate. Of the seven observational studies (8, 9, 13, 14, 16, 17, 22), four used the geometric compensation technique or its modification (8, 9, 16, 22). The main endpoint was related to indications, postoperative complications and cosmesis. **Table 1** summarizes the main published results related to EO. **Supplementary Table 1** shows the main results related to the studies.

**Figure 1:**
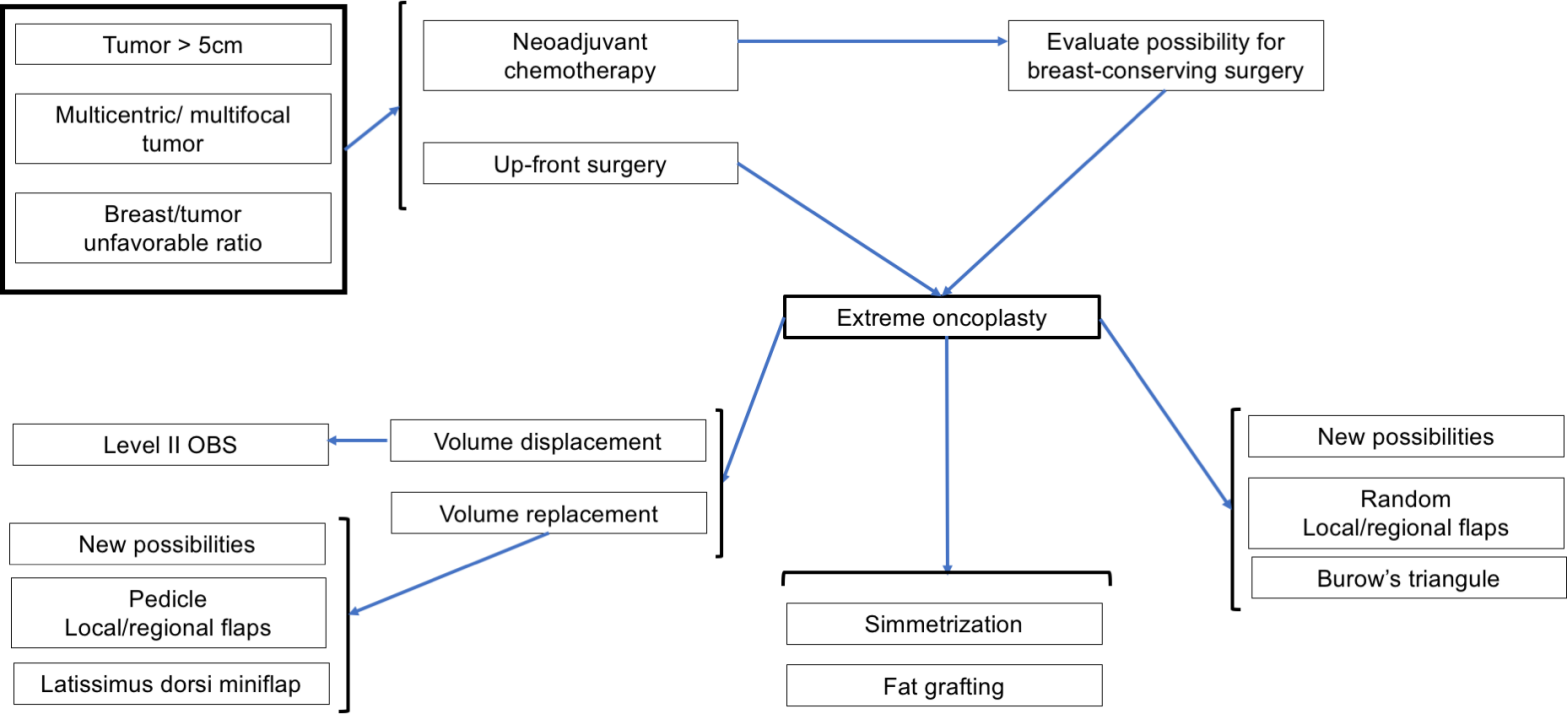
Indications flow associated with Extreme Oncoplasty and associated surgeries. NCT, neoadjuvant chemotherapy.

**Figure 2:**
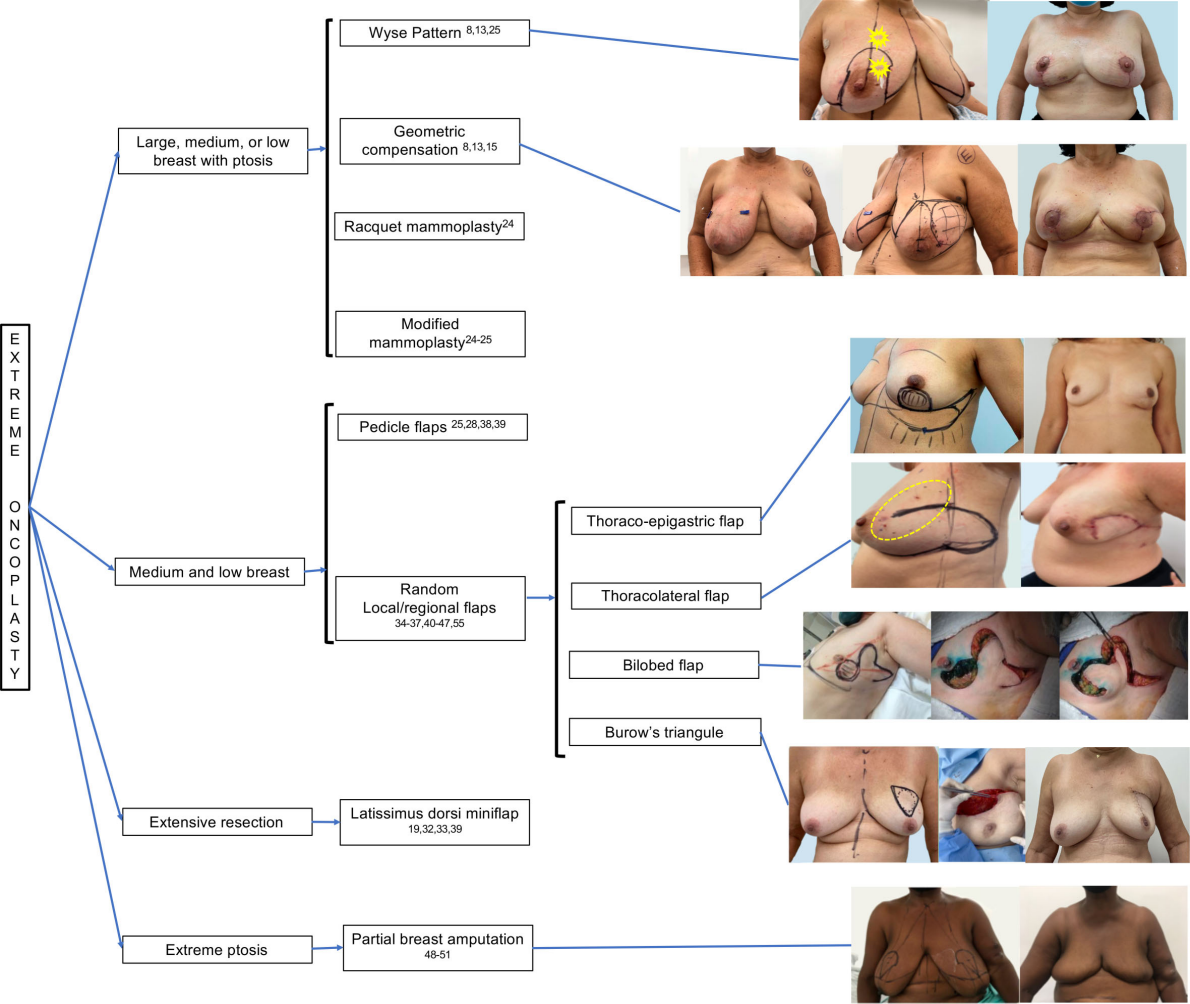
Indications for Extreme Oncoplasty based on breast characteristics.

Retrospective cohort studies maintained the indications for EO (13, 14, 17), showing that it is a safe procedure for large tumours (> 5 cm), multicentric tumours, and multifocal tumours with acceptable complication rates (7.7% to 28%) (8, 9, 13, 14, 17) and low recurrence rates at a follow-up of > 5 years (6% to 9%) (12, 17). Some studies mentioned breast sized/tumour size ratio or resection size to breast size ratio (35, 36), which can be used for small- and medium-sized breasts, using regional tissue transfer with local/regional flaps (18, 35–37). We also observed new options, such as regional flaps (38–47), partial breast amputation (48–51), and flap guides for central tumours (52). It is important to accept and include these new indications in the spectrum of EO. With this in mind, **Table 2** summarizes all possible indications, and **Figures 1** and **2** show the indication flowchart.

The re-excision rate of EO is acceptable (0–12.5%) (7, 8, 12, 14, 15, 17). It was high (37.8%) in a study in which 73.9% of patients had multifocal/multicentric disease (13). The rate of conversion to mastectomy ranges from 0% to 13.5% (8, 13, 14, 34). One study reported 21% and 3% of mastectomy when EO was associated with traditional mammoplasty or latissimus dorsi, respectively (34).

The studies reported different follow-up, which were generally short and approximately 12 (14, 22), 24 (9, 10), and 36 months (8, 13, 16). Four studies had a follow-up higher than 60 months (12, 15, 17, 23).The recurrences were described as locoregional or local recurrences. Although limited follow up, local recurrence reported is lower than 3.5% (14/413 patients). The rate of breast symmetrisation is variable (48–100%) (10, 13, 17), and although it is not part of EO, it allows us to evaluate symmetry and cosmesis. All studies have shown that EO is a safe procedure (7, 12) with acceptable cosmetic results (8, 9, 16).

OS is associated with high satisfaction with the breasts (78%–83.5%) (8, 9, 12, 16, 22) and seems to improve the quality of life (12, 14, 22). Three studies evaluated quality of life using the Breast-Q questionnaire. One, a case series (n=39), reported high (>75%) satisfaction with the breast, outcomes, psychosocial well-being, and sexual well-being (14). The second study reported high scores associated with satisfaction with outcome and satisfaction with breasts (22). The third was a case−control study, which evaluated BCS (n=171) versus OE (n=33) and observed superior scores for OE in the outcome, satisfaction with the nipple complex, and psychological well-being (12).

## Discussion

In 2014, Silverstein et al. (7) presented a new paradigm for OS, putting forth the concept of EO (10). Sixty-six potential candidates for a mastectomy with an unfavourable breast/tumour ratio due to the presence of tumours larger than 5 cm and/or multicentric/multifocal tumours (10) were subjected to standard WP reduction or split reduction procedures and immediate contralateral surgery to achieve symmetry (10). In the same year (2014), another publication showed, through mammaplasty techniques, the achievement of BCS in 17 cases of advanced tumours using the modified WP, called GC (9). This technique emphasizes breast preservation in situations when a breast-versus-tumour relationship is unfavourable for BCS. The skin is resected over the tumour, but using a modification of the standard WP and constituting an EO, which shows satisfactory cosmetic results (9, 28). The procedure is also performed for unicentric tumours < 5 cm with resection of the skin over the tumour, valuing other indications for EO (9), which was also evidenced in a larger series that used split reduction (16). A recent case series with systematic review refined the indications, considering the breast-size ratio, and it showed results associated with small and medium breasts (8). Since the objective of this study was to evaluate the indications, techniques and main results associated with EO, we opted to separately discuss all aspects related to EO, performing an integrative review.

EO is not for beginners (31). For OS, it is necessary to plan, perform clinical and imaging evaluations, and have surgical training (30, 34). Clinical evaluation, preoperative radiological evaluation, intraoperative frozen section margins, intraoperative specimen radiography, and clipped cavity margins are helpful for patient selection and operative evaluation (29, 31). Only one publication has considered the importance of breast nuclear magnetic resonance for surgical planning in the presence of multifocal/multicentric lesions (20).

The EO definition is extended to candidates for mastectomy who underwent BCS (14, 30), including patients with extensive ductal in situ, previously irradiated breast, locally advanced breast carcinoma with limited or partial imaging response to neoadjuvant chemotherapy, past excision biopsy with inappropriate scarring (14), extensive microcalcifications, and an unfavourable tumour/breast volume ratio (8, 21).

With the wide knowledge and dissemination of EO, it is necessary to review potential techniques and group them to facilitate decision-making regarding the indications, surgical possibilities, technical training, and associated complications (8, 18). GC (9) is a technical modification of the initially described procedure (22). An exceptional example of such a literature review was recently published that evaluated patients undergoing GC and WP and examined 243 patients previously described to have undergone this procedure (8). In that review (8), 36 patients were included. The indication for GC was extended to single tumours of smaller size and medium-to-small breasts, provided that they presented with ptosis and that EO was possible in tumours with an unfavourable breast/tumour ratio, valuing the indication for EO in tumours smaller than 5 cm.

When evaluation studies about EO, we have to review the level of oncoplastic surgery. Urban (53) considered three levels of Oncoplasty and EO would be considered for Level II procedures. Clough et al. (3) considered two levels and EO would be considered for Level II: extensive resections, requiring mammoplasty techniques, representing 20-50% of the breast/volume ratio. In 2019, the American Society of Breast Surgeons (6) began to use the term volume displacement and volume replacement. Volume displacement techniques are Level I (< 20%) and Level II techniques (20-50%), and volume replacement (>50%) are local/regional flap reconstruction, miocutaneous flaps and implants. Among the procedures performed for EO, most used WP reduction mastoplasty (8, 9, 17), followed by mastopexy and racquet mammoplasty (13), which is associated with Level II OS procedures associated with volume displacement (6). We have to add volume replacement techniques to arsenal of options related to EO (**Figures 1**, **2**). After reviewing the concept of extensive resection associated with BCS, we found that other techniques could be included, such as pedicled flaps (17) and flaps with lateral thoracic perforators (19). One study compared traditional EO with latissimus dorsi (LD) miniflap (15), with lower complications and higher revision related to LD but no impact on local recurrence.

The EO concept extends the original technique to the use of locoregional flaps (37) and other techniques where extensive resections would lead to loss of cosmetic results (54), while OS allows safe resection with acceptable results. New possibilities for EO are random flaps (55), pedicled flaps (38), latissimus dorsi miniflaps (12, 39), and partial breast amputation (48–51). The techniques are associated with volume replacement (37) for small-to-moderate-sized breasts. For example, of random flaps, we have thoraco-epigastric flap, thoracic-lateral flap, bilobed flap and Burow’s triangle (55), but they can be used in lower resections. Older techniques used before the emergence of OS should not be forgotten. They are usually performed in a nonstandard way for patients in whom preservation of the breast is desired and cosmesis is not the primary endpoint. These techniques are locoregional flaps (37, 38, 40–47, 52, 55) and nonpedicled flaps, such as thoracoepigastric, thoracic-lateral, and bilobed flaps (55). It is necessary to accept and group these new techniques, allowing other reviews in the future.

Articles have shown images of voluminous breasts (7–10, 16, 19, 32) and medium-sized breasts (7, 8) subjected to EO. Hence, we must use techniques such as mammoplasty WP, GC (8, 9, 16, 22), and other mammoplasty techniques (13, 17). Some techniques are associated with volume replacement for small- to moderate-sized breasts (37). The presence of a small-to-moderate-sized nonptotic breast presenting centrally located breast cancer was initially considered a limitation, but perforator flaps are useful in this condition (19).

All patients who are candidates for EO should be aware of the possibility of conversion to mastectomy (8), and skin-preserving mastectomy may eventually be an option. This requires prior reservation of a breast prosthesis if BCS with OS is not safe during surgery based on the tumour margins of breast cosmesis.

Symmetrisation has an unknown impact on quality of life (56) since patients evaluate their cosmesis better than health professionals (57). Another option that can be used after extensive resection associated with BCS is immediate autologous fat grafting (58), which can improve patient selection for EO.

We try to evaluate the quality of the studies, but there was no randomized study and RoB 2.0 assess risk of bias was not performed, and for observational studies we used MINORS (**Supplementary Table 3**) and NOS scores (**Supplementary Table 4**). The major problem observed was too little description of the control group and short follow up in some studies. Although scores are low these studies are important to show the importance of EO. As it is an innovation, the follow up is low and we need more time to evaluate local recurrence. There is a lack of a paired matched case-control study, and new studies need to be performed, comparing EO, oncoplastic surgery and simple breast-conserving surgery. Locorregional recurrence would not be an endpoint but local recurrence. The future authors must take care reporting adequate follow up, loss of patient and local disease-free recurrence.

EO arose due to the need for breast preservation in cases that were difficult to resolve. This fact makes it impossible to carry out prospective randomized studies. It is unethical to perform a mastectomy when breast-conserving treatment can be performed. It limits the quality of the studies (**Supplementary Table 3**). Therefore, we must improve the literature (59), seeking to report the main metrics reported in previous studies (**Supplementary Table 2**), aiming to standardize information. Future studies determine the complexity of performing different procedures, reporting the experience of training centres in oncoplasty, and evaluating the learning curve, mastectomy conversion rate, complications, re-excision rate, local recurrence, patient satisfaction, and cosmetic results of different techniques. Also, it is necessary to perform matched case-control studies, with a long follow-up period.

EO implies developing clinical training to select cases, technical knowledge to evaluate different oncoplastic solutions, a fact that denotes a long learning curve. It is important to decrease barriers to OS (60) if we want to increase the use of EO. When performing OS, it is important to report the indications, type of surgeries (61, 62), postoperative endpoints and long term results (**Supplementary Table 2**). The EO qualifies the service and should be one of the parameters to be used in the quality assessment of breast centres.

Reflections and discussions of published articles (29, 30, 34) are important, but systematic reviews (8) are essential. Since the definition of EO (7, 11), the literature has evolved in indications, and this review considers the new technical possibilities (**Table 2**). Future systematic reviews evaluating the different techniques will facilitate a better understanding of the multiple technical availabilities and results, helping surgical oncologists choose the right procedure for BCS from the multiple techniques of EO.

## Data availability statement

The original contributions presented in the study are included in the article/**Supplementary Material**. Further inquiries can be directed to the corresponding author.

## Author contributions

RV: Conceptualization, Data curation, Formal analysis, Investigation, Methodology, Project administration, Supervision, Visualization, Writing – original draft, Writing – review & editing. IdO-J: Formal analysis, Investigation, Methodology, Visualization, Writing – original draft, Writing – review & editing. RP: Methodology, Visualization, Writing – original draft, Writing – review & editing.
