# Supplementary material for: Extreme oncoplasty: past, present and future
Source: Front Oncol. 2024 Jan 30;13:1215284. doi: 10.3389/fonc.2023.1215284 (PMC10862476; doi:10.3389/fonc.2023.1215284)
Supplement: Supplementary file 1 [file DataSheet_1.pdf]

**EXTREME ONCOPLASTY: PAST, PRESENT, AND FUTURE**

## Supplementary Material

**S. Figure 1.** PRISMA flowchart ........................................................................................ 2

**S. Table 1.** Main Endpoints used in studies reporting Extreme Oncoplasty...... 3

**S. Table 2.** Points to be considered when performing studies about Extreme Oncoplasty........................................................................................................................ 6

**S. Table 3.** Quality of the studies based on MINORS method for non-randomized studies ........................................................................................................................ 8

**S. Table 4.** Quality of the studies based on NOS scale ............................................... 9

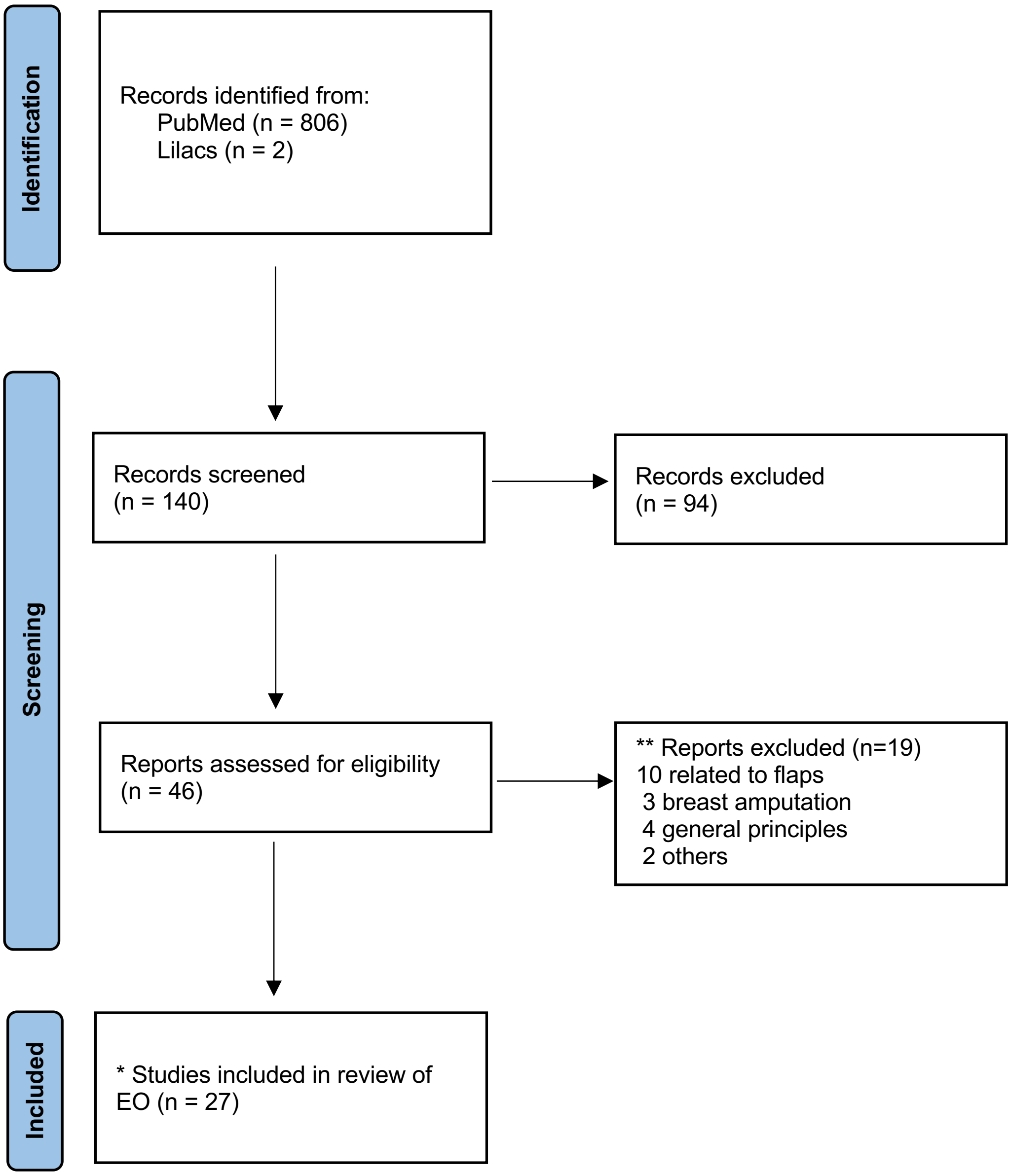


**Supplementary Figure 1.** PRISMA flowchart*

EO= Extreme Oncoplasty; *Main studies reporting EO; **Studies reporting new possibilities associated with EO

**Supplementary Table 1.** Main Endpoints used in studies reporting Extreme Oncoplasty

| Author | Year | Cases | Control | Type | Indications | Main Endpoints |
|---|---|---|---|---|---|---|
| **Case-control Studies** | | | | | | |
| Silverstein (10) | 2015 | 66 | 245 | Case-control<br>EO x OS | Multifocal/multicentric; tumour size > 50mm | - Higher: Median tumor size (77 vs 23mm); free ink tumour (83.3% vs 96%); margin <1mm (54.5% vs 88.6%); rate or conversion to mastectomy (6.1% vs 0,4%)<br>- Equal follow up (24 months: re-excision rate (9.1% vs 6.9%); local recurrence (1.5% vs 1.2%) |
| Acea Nebil (12) | 2017 | 33 | 204 | Case-control<br>EO x OS | Extreme breast conservation | - Equal: age (51 vs 52.5), BMI (27.9 x 26.5), hospital stay (1.74 vs 1.79), reoperation (3.03 vs 2.3%), locoregional recurrence (9.0 vs 9.1%), Physical well-being (91.0 vs 82.1), Sexual well-being (76.2 vs 70.0)<br>- Higher: clinical stage, surgical weight (332.7 vs 200.8g), tumour size (3.01 vs 1.55cm), satisfaction with breast (83.5 vs 76.3)<br>- Follow up 66.6 ±45 months |
| De Lorenzi (23) | 2022 | 100 | 100 | Matched case-control<br>EO x Mastectomy | Multicentric and multifocal tumours<br>Matched: year, age, pT-TNM, multicentric/multifocal tumours | - Higher radiotherapy rate (associated to BCS; 100 vs 50%).<br>- Equal: pN-TNM, grade, luminal subtype, adjuvant systemic therapy<br>- Endpoint 5-year: Equal: Overall Survival (92.5 vs 97.8%), Disease free-survival (81.8 vs 81.8%); local recurrence (1.1 vs 2.2%) |
| Pearce(15) | 2020 | 62LD | 28TM | Subgroup analysis | EO-LD versus EO-TM | -No statistics: surgical complication (18 vs 36%), surgical revision (6 vs 18%), positive margin (8 vs 21%), re-excison (5 vs 0%), mastectomy (3% vs 21%),<br>-Higher: specimen weight (222 x 414g),<br>-Equal: tumor size (68 vs 64mm); multifocal rate (28 vs 29%), 5-year local recurrence-free (100 vs 96.2%) |
| **Observational studies** | | | | | | |

| | | | | | | |
|---|---|---|---|---|---|---|
| Paulinelli (9) | 2014 | 17 | - | Multicentric study | Tumour near the skin. Voluminous malignant tumours in relation to their breast size. Geometric compensation technique | -Mean tumor size 43.6cm; 7 Locally advanced, 3 neoadjuvant chemotherapy; 7 Superior pedicle and 5 inferior pedicle; Ptosis 1 to 3; All free margins; no reoperation, hematoma or dehiscence<br>-Aesthetic result was excellent 7/17 cases. Follow up 28.2 months with no recurrence. |
| Paulinelli(16) | 2020 | 73 | - | Multicentric study | Geometric compensation technique. Locally advanced, multicentric. | -Mean tumor size 30.4mm; 30.7% locally advanced; 20.5% multicentric; 34.4% neoadjuvant chemotherapy; 2.7% of positive margins<br>-Complication: 15,1% wound dehiscence, 12.3% fat necrosis, 6.8% skin necrosis, 9.6% enlarged scar, 2.7% infection<br>-80.8% excellent and good cosmetic results<br>- Follow up 35.3 months: 4.3% of local recurrence |
| Crown(13) | 2019 | 111 | - | Breast unit | Lesions > 5cm; multicentric/multifocal | -73.8% multifocal/multicentric with average of 3.2 lesions per breast; average 57mm<br>-78.3% of no tumour on ink margin. 51.4% of additional surgery for inadequate margins. 12.6% elected to have mastectomy and 37.8% opted for re-excision.<br>-16.2% total complication; 7.2%deishence, 3.6% hematoma, 1.8% cellulitis<br>- Good to excellent cosmetic results were reported in 95% of patients who ultimately achieved breast conservation.<br>- Follow up of 36 months: 3.1% of local recurrence rate |
| Koppliker(14) | 2019 | 39 | - | Breast unit | Lesions > 5cm; multicentric/multifocal; extensive ductal carcinoma in situ | -75mm tumor size, 433g median volume, 43.6% neoadjuvant chemotherapy, 100% free margins; no re-excision, no mastectomy,<br>- No major complications; 7.7% of complications, 5.1% wound dehiscence, 2.6% seroma<br>- Follow up 12 months: no local recurrence<br>- Excellent Breast-Q scores: 78.0 satisfaction with breast |
| Paulinelli(22) | 2021 | 25 | - | Multicentric study | Disguised Geometric Compensation Mammoplasty. Locally advanced and multicentric tumours | - 47.2mm mean size before chemotherapy, 44 locally advanced, 4% multicentric, 36% neoadjuvant chemotherapy<br>-1 (4%) focus of CDIS treated with radiotherapy<br>-6 (24%) minor complication<br>- 16.3 months of follow up: 4% local recurrence<br>-21 (95.5%) cases excellent or good cosmesis<br>-BREAST-Q scores: 81.5 (±15.0) satisfaction with the breasts; 90.4 (±11.7), satisfaction with outcomes |

| | | | | | | |
|---|---|---|---|---|---|---|
| Savioli(17) | 2021 | 50 | - | Breast unit | Lesions > 5cm; multicentric/multifocal | -55mm median tumor size, 44% multifocal disease, specimen weight 243g<br>- 9 (18%) were found to have positive margins; 3 (6%) re-excision rate; 6 (12%) proceeding to mastectomy<br>- different techniques<br>- 28% surgical complications; 10% hematoma; 10% delayed wound healing, 2% cellulitis<br>- 62 months: 10% recurrence rate; no description of local recurrence |
| Franca(8)* | 2022 | 36 | - | Breast unit | Geometric compensation technique | -36mm mean size; 7 (19.4%) small breast with or without ptosis<br>-17.6% neoadjuvant chemotherapy<br>- 69.4% upper pedicle<br>- 8.2% margin lower than 2mm; 5.6% conversion to mastectomy<br>-94.4% synchronous symmetrization<br>-22.2% of postoperative complications: 13.9% skin necrosis, 8.3% dehiscence without delay to start adjuvant therapy<br>-36,6 months of follow up: no recurrence<br>- 84.8% good or reasonable cosmesis |

BCS= breast conserving-surgery; OS= Oncoplastic Surgery; EO= extreme oncoplasty; EO-TM= Extreme Oncoplatic Surgery with therapeutic mammoplasty; EO-LD= Extreme Oncoplasty with Latissimus Dorsi; BMI= Body Mass Index; IORT= intraoperative radiotherapy; SD= significant difference; ND= not significant difference; *Systematic review of the literature submitted to Geometric compensation

**Supplementary Table 2.** Points to be considered when performing studies about Extreme Oncoplasty

| | Point to evaluate | Endpoint to be considered |
|---|---|---|
| Planning | Breast/tumor volume ratio | There is no standard. Selection case by case |
| | | Decrease in breast volume acceptable for breast maintenance, even breast size remains small |
| | Indication | Tumor > 5cm; multicentric/multifocal tumors; unfavorable breast/tumor rate |
| | Breast size | Small, moderate, large |
| | Breast cup | A to D |
| | Ptosis | Grade |
| | Radiology | Minimum: mammography, breast ultrasound; Nuclear resonance (optional) |
| | Tumor size | Total; Invasive/ DCIS; Tumor size before and after NCT; |
| | Multifocal disease | Number of tumors; mean multifocal size |
| | Neoadjuvant chemotherapy (NCT) | There is no standard for radiological evaluation. Selection case by case. |
| Surgery | Volume displacement/ Level II oncoplasty | Wise pattern mammoplasty |
| | | Geometric compensation/Disguised geometric compensation |
| | | Other mammoplasty techniques |
| | | Partial breast amputation (new) |
| | | Grisotti/ other central techniques (new) |
| | Volume replacement (new) | Miocutaneous flaps: latissimus dorsi, TRAM (new) |
| | | Pedicle flaps (new) |
| | | Random flaps (new) |
| | Resected volume | g |
| | Upfront symmetrization | Absent/ present. |
| | Margin | % of Free margin; % or re-excision; CDIS or invasive margin |
| | Pedicle used | Superior, inferior, medial, lateral, multiple |
| | Conversion to mastectomy | % of conversion |
| | Surgeon training | Learning curve (perspective) |
| | Metal clip | Tumor bed for radiotherapy (perspective) |

| | Point to evaluate | Endpoint to be considered |
|---|---|---|
| Postoperative | Complication rate | Types: Hematoma, infection, dehiscence, skin necrosis, areola necrosis, seroma, enlarged scar |
| | | Rate of total complication (percentage of patients by procedure) |
| | | Simple (each complication); Minor or major complication |
| | | Time to send for the next treatment (radiotherapy or chemotherapy) |
| | | Surgery because early complication |
| | Complication evaluation (perspective) | Prolonged seroma > 30 days |
| | | Necrosis/ Dehiscence: Control with debridement and re-suturing |
| | | Classification: Clavien-Dindo; Accordion classification |
| | | Side: local/systemic |
| | Long term (> 1 year) | Fat necrosis, asymmetry |
| | | Lent/Soma for radiotherapy effects (perspective) |
| | Cosmetic results | BBCTcore; Garbay, Fituosi, Harris |
| | Radiotherapy | Boost, metal clips, IORT |
| Long term | Follow up | Mean; 5-year event |
| | Local recurrence | Rate: surgical scar, skin or subcutaneous |
| | | Second ipsilateral breast cancer |
| | | Disease free local recurrence; hazard risk of recurrence |
| | Second breast procedures | % of defect correction or symmetrization |
| | Quality of life | PROMS: BCTOS, Breast-Q |
| Breast Center | Measures of quality control | Rate of extreme oncoplasty; mastectomy conversion rate |

**Supplementary Table 3.** Quality of the studies based on MINORS method for non-randomized case-control studies(24)*.

| | | Silverstein 2015(10) | Acea Nebil 2017(12) | De Lorenzi 2022(23) |
|---|---|---|---|---|
| Item | Information | | | |
| 1 | A stated aim of the study | 2 | 2 | 2 |
| 2 | Inclusion of consecutive patients | 2 | 1 | 2 |
| 3 | Prospective collection of data | 1 | 1 | 1 |
| 4 | Endpoint appropriate to the study aim | 2 | 2 | 2 |
| 5 | Unbiased evaluation of endpoints | 0 | 0 | 2 |
| 6 | Follow-up period appropriated to the major endpoint | 1 | 2 | 2 |
| 7 | Loss to follow up not exceeding 5% | 0 | 0 | 0 |
| And in the case of comparative studies | | | | |
| 8 | A control group having the gold standard intervention | 2 | 2 | 2 |
| 9 | Contemporary groups | 2 | 0 | 2 |
| 10 | Baseline equivalence of groups | 0 | 0 | 2 |
| 11 | Prospective calculation to the sample size | 0 | 0 | 0 |
| 12 | Statistical analyses adapted to the study design | 2 | 2 | 2 |
| | Total | 14 | 12 | 19 |

Score: (0) not reported; (1) Reported but inadequate; (2) Reported and adequate.

* One study(15) evaluate two types of EO and was not considered for this evaluation

**Supplementary Table 4.** Quality of the studies based on NOS scale(25)

| | Author | Year | 1 | 2 | 3 | 4 | 5 | 6 | 7 | 8 | Score | Prospective |
|---|---|---|---|---|---|---|---|---|---|---|---|---|
| Retrospective case-control | Silvestein(10) | 2015 | 0 | 1 | 0 | 1 | 1 | 1 | 1 | 0 | 5/9 | - |
| | Acea Nebil(10) | 2017 | 0 | 1 | 0 | 1 | 1 | 1 | 1 | 0 | 5/9 | - |
| | De Lorrenzi(23)* | 2022 | 0 | 1 | 0 | 1 | 2 | 2 | 1 | 0 | 7/9 | - |
| | Pearce(15) | 2022 | 0 | 1 | 0 | 1 | 1 | 1 | 1 | 0 | 5/9 | - |
| Retrospective cohort | Crown(13) | 2019 | 0 | 0 | 1 | 1 | 0 | 1 | 0 | 0 | 3/9 | - |
| | Paulineli(16) | 2020 | 0 | 0 | 1 | 1 | 0 | 1 | 1 | 1 | 5/9 | - |
| | Savioli(17) | 2021 | 0 | 0 | 1 | 1 | 0 | 1 | 1 | 0 | 4/9 | - |
| Prospective cohort | Paulinelli(9) | 2014 | 0 | 0 | 2 | 1 | 0 | 2 | 0 | 1 | 6/9 | Cosmesis |
| | Paulinelli(22) | 2021 | 0 | 0 | 2 | 1 | 0 | 2 | 0 | 1 | 6/9 | PROM + Cosmesis |
| Retrospective + Prospective | Koppliker(14) | 2019 | 0 | 0 | 2 | 1 | 0 | 2 | 0 | 1 | 6/9 | PROM |
| | Franca(8) | 2022 | 0 | 0 | 2 | 1 | 0 | 2 | 0 | 1 | 6/9 | Cosmesis |

NOS scale: A) Selection domain: 1= representativeness of the exposed cohort; 2= selection of the non-exposed cohort; 3= ascertainment of exposure; 4= outcome of interest was not present at start of study; B) Comparability: 5= comparability of cohorts on the basis of the design or analysis; C) Exposure: 6= assessment of outcome; 7= follow-up long enough for outcome to occur; 8= adequacy of follow up. *Matched study.
